# Prognostic Value of Baseline Sarcopenia and Adipose Tissue Indices in HR+/HER2− Metastatic Breast Cancer Treated with CDK4/6 Inhibitors: A Retrospective Cohort Study

**DOI:** 10.3390/jcm15041623

**Published:** 2026-02-20

**Authors:** Latif Karahan, Arif Akyildiz, Taha Koray Sahin, Mustafa Arda Batu, Cagatay Ersan, Mehmet Ruhi Onur, Sercan Aksoy, Deniz Can Guven

**Affiliations:** 1 Department of Medical Oncology, Hacettepe University Faculty of Medicine, 06230 Ankara, Turkey; 2 Department of Radiology, Hacettepe University Faculty of Medicine, 06230 Ankara, Turkey

**Keywords:** metastatic breast cancer, CDK4/6 inhibitors, sarcopenia, adipose tissue, body composition, progression-free survival

## Abstract

**Background/Objectives**: Sarcopenia, defined by reduced skeletal muscle mass, may have prognostic relevance in metastatic breast cancer. Muscle quality, reflected by adipose tissue indices, could also influence outcomes, but evidence in CDK4/6 (cyclin-dependent kinase)-inhibitor-treated patients is limited. We therefore evaluated the prognostic impact of baseline sarcopenia and adipose tissue distribution indices in this population. **Methods**: We retrospectively analyzed 156 women with HR+/HER2− MBC (hormone-receptor-positive, Her2-negative metastatic breast cancer) who initiated ribociclib or palbociclib plus endocrine therapy between May 2020 and January 2024. Association between L3 computed tomography (CT)-derived skeletal muscle index (SMI) and adipose tissue indices was evaluated with univariable and multivariable analyses. Sarcopenia was defined as SMI < 41 cm^2^/m^2^. **Results**: Median age was 57.6 years; 75% of patients were postmenopausal, and 48% of the cohort were sarcopenic. Median progression-free survival (PFS) for the entire cohort was 24.7 months (95% CI: 20.3–29.2). Patients with baseline sarcopenia had substantially shorter PFS compared to those without sarcopenia (21.5 months (95% CI: 10.9–32.1), versus 27.1 months (95% CI: 15.2–39; *p* = 0.016). Multivariable Cox regression analyses identified two independent predictors of prolonged PFS: non-sarcopenia (SMI ≥ 41 cm^2^/m^2^) and de novo metastatic disease. BMI (body mass index) and all adipose indices were not associated with PFS. **Conclusions**: Baseline non-sarcopenia and de novo metastatic disease independently predict longer PFS on CDK4/6 inhibitors, whereas adiposity measures and BMI are not prognostic. Routine body composition assessment may refine risk stratification and identify candidates for supportive interventions. Prospective studies are needed to validate these findings.

## 1. Introduction

Breast cancer is the most commonly diagnosed malignancy in women worldwide and the second leading cause of cancer mortality [1]. Approximately 60–70% of breast cancers are hormone-receptor-positive (HR+) and human-epidermal-growth-factor-receptor-2-negative (HER2−) [2]. For patients with advanced or metastatic HR+/HER2− breast cancer (MBC), endocrine therapy is the backbone of treatment; however, the emergence of endocrine resistance is a major therapeutic challenge [2]. In recent years, the addition of CDK4/6 inhibitors to endocrine therapy has become a standard first-line treatment in this setting, yielding substantial prolongation of progression-free survival (PFS) by several months compared to endocrine therapy alone [3]. Three CDK4/6 inhibitors (palbociclib, ribociclib, abemaciclib) have demonstrated significant efficacy in combination with endocrine therapy, improving clinical outcomes and quality of life in HR+ MBC [4,5,6]. Despite these advances, there remains heterogeneity in patient outcomes, prompting interest in host factors—such as body composition—that might influence treatment efficacy and prognosis.

Body composition, referring to the distribution of lean mass and adipose tissue in the body, has emerged as an important prognostic factor in oncology [7]. Traditional metrics like body mass index (BMI) are of limited utility, as they do not distinguish between muscle and fat; for instance, an obese patient with significant muscle wasting (“sarcopenic obesity”) may have a normal or high BMI that masks underlying frailty [8]. More precise analysis is obtained via computed tomography (CT) imaging of cross-sectional muscle and fat areas. Low skeletal muscle mass, or sarcopenia, is common in cancer patients as a result of tumor-associated cachexia and age-related muscle loss, and it has been linked to poorer outcomes across multiple tumor types [7]. Meta-analyses indicate that sarcopenia portends worse overall survival (OS) in cancer patients generally and shorter PFS in patients with metastatic disease [7,9,10]. Beyond muscle mass, visceral adipose tissue is an active endocrine and inflammatory organ that may influence systemic therapy outcomes through metabolic and cytokine/adipokine signaling. Accordingly, quantifying adipose compartments alongside skeletal muscle may provide prognostic information beyond BMI [11].

In breast cancer, evidence is growing that body composition at diagnosis or treatment initiation can affect prognosis. Caan et al. analyzed 3241 patients with non-metastatic breast cancer and found that sarcopenia (present in 34% at diagnosis) was associated with a 41% higher risk of overall mortality [11]. Notably, patients with combined high adiposity and sarcopenia had the worst survival, while BMI alone was not prognostic [11]. Likewise, in early-stage breast cancer treated with curative intent, low muscle mass and muscle fat infiltration have been identified as independent predictors of shorter disease-free and OS [8]. These data underscore that beyond obesity, the quality and quantity of muscle are critical factors influencing breast cancer outcomes.

In the metastatic setting, sarcopenia is common and may adversely affect outcomes by reflecting cancer-related catabolism and reduced physiologic reserve [12]. Several retrospective studies suggest that low muscle mass is associated with inferior PFS and OS in metastatic breast cancer patients treated with CDK4/6 inhibitors, whereas higher visceral adiposity may correlate with improved outcomes [13,14]. Notably, Cavdar et al. reported significantly shorter PFS and OS in sarcopenic patients receiving CDK4/6 inhibitors, without increased treatment-related toxicity [13]. Similar associations have been observed in other single-center cohorts [14,15].

Despite these findings, evidence remains limited and heterogeneous. A recent systematic review confirmed a high prevalence of sarcopenia in MBC but did not demonstrate a consistent prognostic impact on survival, likely due to heterogeneity in sarcopenia definitions, patient populations, and treatment settings [7]. Moreover, data focusing on CDK4/6-inhibitor-treated patients, particularly incorporating muscle quality and adipose tissue indices as markers of body composition, are scarce.

Taken together, the literature indicates that baseline body composition—particularly skeletal muscle mass—may be a critical modifier of outcomes in HR+ MBC, especially in the era of CDK4/6 inhibitors. Nevertheless, data remain somewhat limited and occasionally conflicting regarding the role of adipose tissue distribution on efficacy and survival. To address these gaps, we conducted a retrospective study in a cohort of HR+/HER2− MBC patients treated with CDK4/6 inhibitors plus endocrine therapy. Our aims were to determine the prevalence of baseline sarcopenia in this cohort; to evaluate the association of sarcopenia with treatment outcomes, including response, and PFS; and to explore whether quantitative indices of adipose tissue stratify patients’ outcomes. We hypothesized that sarcopenia at treatment initiation would be associated with worse PFS, independent of BMI, and that extreme body composition phenotypes (e.g., sarcopenic vs. non-sarcopenic obesity) might differentially affect outcomes. By elucidating these relationships, our study seeks to inform risk stratification and encourage integration of body composition assessment into the management of MBC.

## 2. Materials and Methods

### 2.1. Study Population and Design

This study was a retrospective cohort of patients with metastatic breast cancer treated at a single tertiary oncology center. We included female patients (age ≥ 18) with HR-positive, HER2-negative metastatic or locally advanced recurrent breast cancer who initiated therapy with a CDK4/6 inhibitor (ribociclib or palbociclib) in combination with endocrine therapy (aromatase inhibitor or fulvestrant) between May 2020 and January 2024, and who had an evaluable baseline abdominal CT or PET-CT scan performed within 30 days prior to treatment initiation for body composition analysis.

Patients who received CDK4/6 inhibitors as either first line or second line (after progression on one prior endocrine therapy) were eligible. We excluded patients with HER2-positive disease, those without a suitable baseline CT scan performed within 30 days prior to treatment initiation for body composition analysis, patients with an Eastern Cooperative Oncology Group performance status (ECOG-PS) of 2–3, male breast cancer cases, patients with less than 6 months of follow-up, and those with other malignancies or conditions precluding assessment of outcomes.

Baseline patient and disease characteristics, including age, menopausal status, ECOG-PS, comorbidities (e.g., diabetes mellitus), metastatic sites, and prior treatments, were collected from electronic medical records. Tumor receptor statuses were collected by pathology reports (estrogen and/or progesterone receptor ≥1% defined HR-positive; HER2-negative defined as immunohistochemistry score 0 or 1+, or 2+ with FISH-negative). The study was conducted in accordance with the Declaration of Helsinki and was approved by the Institutional Ethics Committee (approval number: 2024/07-42).

### 2.2. Body Composition Measurement

**Figure 1:**
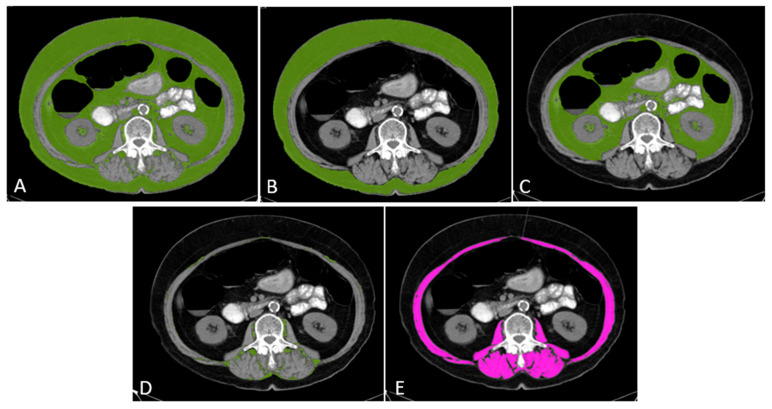
The TAT, SAT, VAT and IMAT areas are highlighted in green (**A**–**D**). SMA was calculated as the summed cross-sectional area of the psoas, abdominal wall, and paraspinal muscles, highlighted in pink (**E**). **Abbreviations:** TAT: Total adipose tissue, SAT: Subcutaneous adipose tissue, VAT: Visceral adipose tissue, IMAT: Intermuscular adipose tissue, SMA: Skeletal muscle area.

For each patient, a diagnostic computed tomography (CT) scan of the abdomen or positron emission tomography CT (PET-CT) scan obtained at baseline (within 30 days before starting the CDK4/6 inhibitor) was used for body composition analysis. A single axial image at the level of the third lumbar vertebra (L3) was analyzed, as this level correlates with whole-body lean and fat mass [8,16]. Trained radiologists used Syngo.via software (Siemens Healthcare, Erlangen, Germany; VC10C) to delineate skeletal muscle and adipose tissue cross-sectional areas. Skeletal muscle area (SMA) was quantified by summing the cross-sectional area of all musculature at L3 (including psoas, erector spinae, quadratus lumborum, transverse abdominis, external and internal obliques, and rectus abdominis muscles) within a predefined Hounsfield unit (HU) range for lean muscle (approximately −29 to +150 HU). Visceral adipose tissue (VAT) area was measured within the peritoneal cavity (excluding vertebral and paraspinal muscles) using a typical fat attenuation range (−190 to −30 HU), while subcutaneous adipose tissue (SAT) area was measured between the skin and musculature at the same level. Intermuscular adipose tissue (IMAT), representing fatty infiltration within muscle, was captured either as part of muscle HU distribution or segmented if distinguishable. Total adipose tissue (TAT) area was calculated as the sum of VAT, SAT, and IMAT areas (in cm^2^). Each area measurement was normalized to patient height in meters squared (m^2^) to derive the following indices: skeletal muscle index (SMI = SMA/height^2^), VAT index (VAT/height^2^), SAT index (SAT/height^2^), IMAT index, and TAT index (TAT/height^2^) [8]. The measurement of SMI and adipose tissue indices is illustrated in Figure 1.

All segmentations were performed by trained radiologists using a standardized protocol. Segmentations underwent internal quality control; when an initial segmentation required refinement to meet prespecified quality criteria, the measurement was repeated by a second radiologist, and a third senior radiologist reviewed and adjudicated the final segmentation. Measurements were performed within routine clinical workflow, and formal blinding was not implemented. Formal interobserver reproducibility metrics (e.g., intraclass correlation coefficients) were not assessed.

We defined sarcopenia based on low SMI using sex-specific cut-offs from the oncology literature. In this study, sarcopenia was operationalized as CT-defined low skeletal muscle mass based on SMI cut-offs, rather than a functional sarcopenia diagnosis. Muscle strength or physical performance measures (e.g., handgrip strength, gait speed) were not available in the dataset. For women, an SMI < 41 cm^2^/m^2^ is a widely accepted threshold for sarcopenia (originally derived from cohorts of cancer patients) [17,18,19]. Patients in our study with SMI < 41 were categorized as sarcopenic, and those with SMI ≥ 41 as non-sarcopenic. In contrast to muscle mass, there are no established consensus cut-points for defining “high” versus “low” adipose tissue indices in this population. Therefore, adipose tissue indices were dichotomized according to their median values within the study cohort. Patients were classified into “high” and “low” groups for VAT, SAT, IMAT, and TAT indices based on the respective median values. Baseline BMI was calculated as weight (kg) divided by height squared (m^2^) and categorized according to World Health Organization criteria, with BMI ≥ 30 kg/m^2^ defined as obesity.

### 2.3. Treatment and Follow-Up

All patients received a CDK4/6 inhibitor (ribociclib, *n* = 117; palbociclib, *n* = 39) in combination with either letrozole (*n* = 84) or fulvestrant (*n* = 72), based on physician discretion and menopausal status. Ribociclib was given at 600 mg daily (3 weeks on, 1 week off) and palbociclib at 125 mg daily (3 weeks on, 1 week off), alongside continuous endocrine therapy. Dose adjustments were made as needed for toxicity per standard guidelines. Patients were assessed clinically and radiologically every ~8–12 weeks. Tumor response evaluations were extracted from available radiology reports and categorized by the treating physicians as objective response, disease stabilization, or disease progression based on routine clinical assessment. For this analysis, PFS was defined as the time from initiation of CDK4/6 inhibitor to the first documentation of disease progression or death from any cause, whichever occurred first. Patients without an event were censored at the date of last follow-up (data cutoff: 1 May 2024).

### 2.4. Statistical Analysis

Descriptive statistics were used to summarize baseline patient characteristics. Continuous variables were reported as median (IQR) or mean ± SD, and categorical variables as counts and percentages. Between-group comparisons (sarcopenic vs. non-sarcopenic) used the chi-square or Fisher’s exact test for categorical variables and Student’s *t*-test or Mann–Whitney U test for continuous variables.

PFS was estimated using the Kaplan–Meier method and compared with the log-rank test. Cox proportional hazards models were used to evaluate associations between body composition parameters and PFS. Variables were screened in univariable Cox analyses, and those with *p* < 0.20 were entered into a multivariable Cox model using a backward stepwise likelihood ratio approach, with sarcopenia retained and additional covariates included to adjust for confounding (e.g., age, line of therapy, visceral metastases). Hazard ratios (HRs) with 95% CIs were reported. An exploratory subgroup analysis stratified by sarcopenia assessed whether adipose tissue indices were associated with PFS within sarcopenic and non-sarcopenic patients. All tests were two-sided with *p* < 0.05 considered significant. Analyses were performed using SPSS v27.0 (IBM Corp., Armonk, NY, USA).

During manuscript preparation, the authors used ChatGPT (OpenAI; GPT-5.2) solely to improve language clarity and correct grammar; the tool was not used to generate or analyze data, perform statistical analyses, or influence study design, results, or conclusions.

## 3. Results

### 3.1. Patient Characteristics

**Figure 2:**
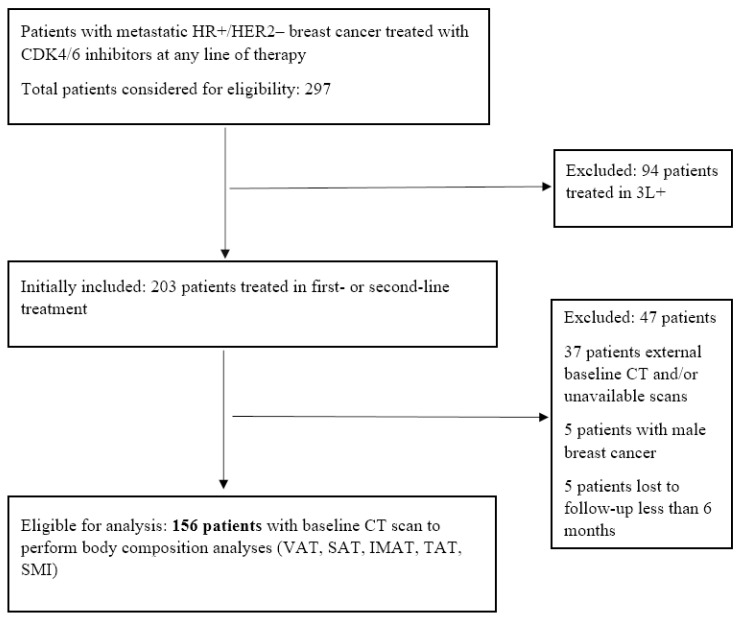
The flow chart of the study population and patient selection. **Abbreviations:** CDK4/6, cyclin-dependent kinase 4/6; CT, computed tomography; HER2, human epidermal growth factor receptor 2; HR, hormone receptor; IMAT, intermuscular adipose tissue; SAT, subcutaneous adipose tissue; SMI, skeletal muscle index; TAT, total adipose tissue; VAT, visceral adipose tissue; 3L+, third-line or later.

A total of 156 patients met the inclusion criteria. The flow chart is shown in Figure 2. The median age at initiation of CDK4/6 inhibitor therapy was 57.6 years (IQR, 48.4–67.6), and 75% of patients were postmenopausal. A total of 50 patients (32.1%) had de novo Stage IV disease. Regarding metastatic sites, 67 patients (43%) had metastases confined to bone-only (±lymph nodes, no visceral organs) and 89 (57%) had visceral metastases (with or without bone involvement).

At initiation of CDK4/6 inhibitor therapy, 80 patients (51.3%) received treatment in the first-line setting for metastatic disease, while 76 (48.7%) were treated in the second line after progression on one prior endocrine therapy. Ribociclib was the most frequently used CDK4/6 inhibitor (*n* = 117, 75%), followed by palbociclib (*n* = 39, 25%). Concomitant endocrine therapy consisted of letrozole in 84 patients (53.8%) and fulvestrant in 72 (46.2%). Baseline ECOG-PS was evenly distributed, with ECOG 0 in 78 patients (50%) and ECOG 1 in 78 (50%). The mean BMI was 27.7 ± 5.0 kg/m^2^, with 47 patients (30.1%) classified as obese (BMI ≥ 30 kg/m^2^). Diabetes mellitus was present in 53 patients (34%). Baseline demographic and clinical characteristics are summarized in Table 1.

Baseline body composition parameters, including skeletal muscle area (SMA) index and adipose tissue indices (SAT, VAT, IMAT, and TAT), are summarized in Table 2. Importantly, nearly half of the cohort had evidence of low muscle mass at baseline: 75 patients (48.1%) were sarcopenic (SMI < 41 cm^2^/m^2^) by our predefined criteria. There were no significant differences between sarcopenic vs. non-sarcopenic groups in distribution of de novo vs. recurrent disease, visceral metastasis incidence (~60% in both), line of therapy, or HER2-low status (all *p* > 0.1). BMI was significantly lower in the sarcopenic group: mean BMI 24.8 ± 3.6 in sarcopenic vs. 30.5 ± 4.5 in non-sarcopenic patients (*p* < 0.001). 20% of sarcopenic patients were actually classified as overweight or obese by BMI. Approximately 18% of sarcopenic patients also had low adipose stores (both low VAT and low SAT indices), a phenotype consistent with cancer cachexia, whereas others had low muscle but high fat.

### 3.2. Treatment Efficacy and Survival Outcomes

All patients were evaluable for response and survival analysis. By the time of analysis, 99 patients (63.5%) had experienced disease progression and 52 (33.3%) had died. The remaining patients were censored (alive without progression for PFS, or alive for OS) at last follow-up.

### 3.3. The Association Between Body Composition Parameters and Progression-Free Survival

**Figure 3:**
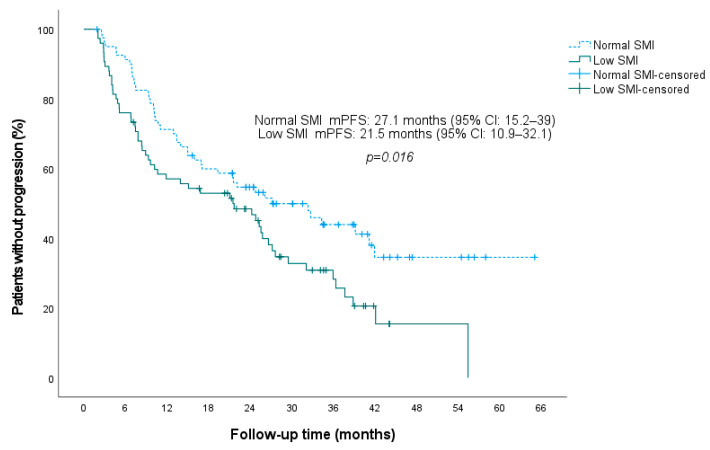
Kaplan–Meier plot of progression-free survival stratified by baseline sarcopenia status. **Abbreviations:** CI, confidence interval; mPFS, median progression-free survival; SMI, skeletal muscle index.

For the entire cohort, the median PFS was 24.7 months (95% CI: 20.3–29.2). We next examined outcomes stratified by baseline sarcopenia status. Patients with baseline sarcopenia had substantially shorter PFS compared to those without sarcopenia. The median PFS for sarcopenic patients was 21.5 months (95% CI: 10.9–32.1), versus 27.1 months (95% CI: 15.2–39) for non-sarcopenic patients (*p* = 0.016; log rank test) (Figure 3).

**Figure 4:**
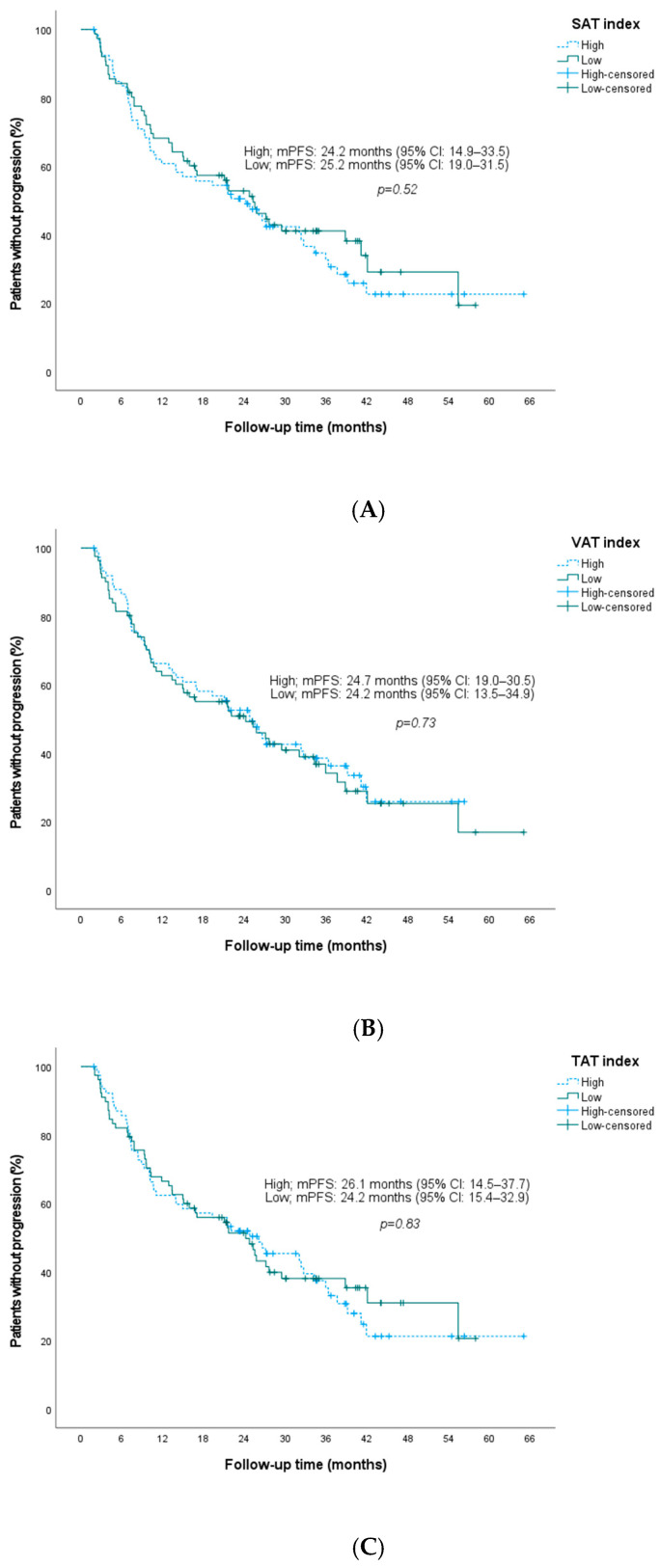
Kaplan–Meier plots of progression-free survival stratified by baseline SAT (**A**), VAT (**B**), and TAT (**C**) indices. **Abbreviations:** SAT, subcutaneous adipose tissue; VAT, visceral adipose tissue; TAT, total adipose tissue; mPFS, median progression-free survival; CI, confidence interval.

**Figure 5:**
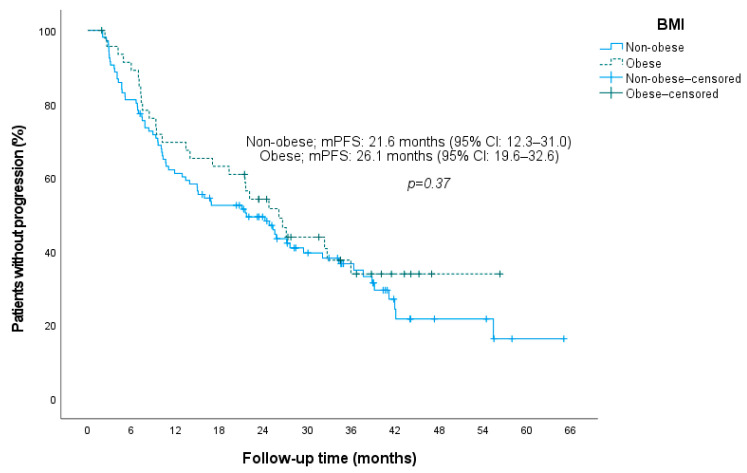
Kaplan–Meier plot of progression-free survival stratified by BMI. **Abbreviations:** BMI, body mass index; mPFS, median progression-free survival; CI, confidence interval.

In addition to sarcopenia, we evaluated adipose tissue indices and BMI in relation to outcomes. Patients were categorized into higher versus lower VAT, SAT, IMAT, and TAT index groups based on cohort-specific median cut-off values (Table 2). None of the adiposity measures were significantly associated with PFS (Figure 4). Consistently, when adipose tissue indices were modeled as continuous variables, none were significantly associated with PFS: SAT index (HR per 1 cm^2^/m^2^ increase: 1.001, 95% CI 0.996–1.007; *p* = 0.650), VAT index (HR: 0.998, 95% CI 0.991–1.004; *p* = 0.447), IMAT index (HR: 0.998, 95% CI 0.936–1.065; *p* = 0.962), and TAT index (HR: 1.000, 95% CI 0.997–1.003; *p* = 0.901). PFS also did not differ significantly between obese (BMI ≥ 30 kg/m^2^) and non-obese patients (*p* = 0.37) (Figure 5).

### 3.4. Univariable and Multivariable Cox Regression Analyses for Progression-Free Survival

Variables with *p* < 0.20 in the univariable Cox regression analysis were entered into the multivariable model. Given that adipose tissue indices (VAT, SAT, IMAT, and TAT) did not demonstrate a significant association with PFS in the analyses described above, these parameters were not carried forward to univariable or multivariable Cox modeling; sarcopenia was retained as the primary body composition exposure. Univariable Cox regression analyses were performed for the following covariates: sarcopenia (yes/no), de novo metastatic disease (yes/no), CDK4/6 inhibitor regimen (ribociclib vs. palbociclib), menopausal status (pre/perimenopausal vs. postmenopausal), ECOG-PS (0 vs. 1), and metastatic pattern (bone-only vs. visceral involvement).

In the multivariable Cox regression, two factors remained independently associated with improved PFS: non-sarcopenia (SMI ≥ 41 cm^2^/m^2^), and de novo metastatic disease. Univariable and multivariable Cox regression analyses results are presented in Table 3.

**Overall Survival:** OS analyses across selected covariates—including sarcopenia, treatment type, ECOG-PS, endocrine partner, age, and menopausal status—did not yield statistically significant associations. At the time of analysis, overall survival data were not yet mature and were therefore not included in the statistical analyses.

## 4. Discussion

CDK4/6-inhibitor–based endocrine therapy remains the standard first-line strategy for HR+/HER2− metastatic breast cancer in contemporary guidelines and pivotal trials [4,5,20,21,22]. In this single-center retrospective cohort treated with CDK4/6 inhibitors, we found that baseline sarcopenia was highly prevalent (48% of patients) and associated with shorter PFS, and importantly, non-sarcopenia (SMI ≥ 41 cm^2^/m^2^) remained an independent predictor of improved PFS after adjustment. These results support our initial hypothesis and corroborate a growing body of evidence that low skeletal muscle mass portends poorer outcomes in patients receiving therapy for advanced breast cancer [13,14,15]. In contrast, adipose tissue indices (VAT, SAT, IMAT, TAT) and BMI were not prognostic for PFS. Alongside body composition, de novo metastatic presentation independently predicted longer PFS, suggesting that host factors and disease biology at metastatic diagnosis both contribute meaningfully to outcomes under CDK4/6 inhibition [23]. Overall, our findings highlight the prognostic importance of muscle mass in the era of CDK4/6 inhibitors and raise important questions about how best to manage sarcopenic patients.

Our sarcopenia findings are consistent with several prior studies. Franzoi et al. reported inferior outcomes among patients with low muscle mass treated with CDK4/6 inhibitors and also highlighted that CT-derived body composition can be feasibly incorporated into routine oncologic imaging workflows [14]. Similar associations between sarcopenia and worse outcomes have been observed in other real-world cohorts treated with CDK4/6 inhibitors, including studies reporting shorter PFS among sarcopenic patients [13,15]. Kripa et al. additionally suggested that lower muscle metrics may relate to inferior treatment response under CDK4/6-inhibitor-based therapy, supporting the concept that muscle depletion may reflect reduced physiologic reserve relevant to treatment efficacy and durability [24]. We note that our sarcopenia definition reflects an anatomical, imaging-based construct rather than sarcopenia as defined by contemporary consensus frameworks (e.g., EWGSOP2), which emphasize both muscle quantity and function [25]. Therefore, our findings should be interpreted as the prognostic impact of low skeletal muscle mass, and future prospective studies incorporating functional measures are warranted. We also acknowledge that CT-defined low muscle mass may partially capture unmeasured disease severity/systemic catabolism; as detailed tumor-burden metrics were not uniformly available, residual confounding cannot be excluded.

OS analyses were not performed because OS data were not sufficiently mature at the time of analysis; accordingly, our conclusions are restricted to PFS. Although some prior cohorts have described an association between sarcopenia and OS, differences in follow-up duration, subsequent therapies, and sarcopenia definitions/prevalence likely contribute to heterogeneous findings [13]. In line with this variability, a recent systematic review and meta-analysis by Jang et al. did not demonstrate a consistent association between sarcopenia and OS in MBC overall [7]. Continued follow-up of our cohort is planned to enable future OS analyses.

In our cohort, baseline adipose tissue indices and BMI were not prognostic for PFS, unlike some prior CDK4/6 inhibitor cohorts suggesting a potential benefit with higher visceral adiposity or obesity [14,15,24]. These discrepancies may reflect differences in cohort composition (e.g., treatment line, metastatic burden/sites, comorbidities), how adiposity is operationalized (area/index and fat quality), and the lack of validated adiposity cut-points—where pragmatic median splits can reduce group contrast and attenuate modest effects. Moreover, muscle and fat are closely intertwined; the sarcopenic obesity literature highlights that excess adiposity may mask clinically meaningful muscle depletion, with muscle loss often dominating risk even in heavier patients [17,26]. Overall, our findings suggest that baseline muscle mass is the more informative host factor for PFS under CDK4/6 inhibition, whereas baseline adiposity indices did not add prognostic information in this cohort. We also observed that de novo metastatic disease independently predicted improved PFS. Prior population and multi-center studies have shown that de novo metastatic breast cancer can have more favorable outcomes than early recurrent metastatic disease, plausibly due to therapy-naïve tumor biology, less endocrine resistance, and differences in metastatic burden or patterns at presentation [23]. In our dataset, the persistence of a de novo advantage after adjustment supports the idea that disease trajectory before metastatic diagnosis remains clinically relevant even in the CDK4/6 era.

From a clinical perspective, our findings emphasize a few key points. First, routine assessment of body composition at baseline could be valuable for patients with metastatic breast cancer. CT-based quantification at standard anatomic levels is feasible and can be derived from imaging already performed for staging and follow-up [27]. Identification of sarcopenia might prompt closer monitoring and perhaps earlier intervention. They might benefit from more aggressive supportive care. For instance, interventions such as nutritional supplementation, resistance exercise programs, and anabolic strategies could be implemented. Whether such interventions can translate into longer PFS in MBC is not yet proven, but trials are warranted.

This study has strengths including a relatively large single-center cohort uniformly treated with CDK4/6-inhibitor-based endocrine therapy and detailed CT-derived body composition assessment. Limitations include the retrospective design and the potential for selection bias related to imaging availability. We did not capture longitudinal body composition changes or direct functional measures (e.g., grip strength); accordingly, we could not apply consensus sarcopenia definitions requiring functional assessment (e.g., EWGSOP2) and OS analyses were not performed because OS data were not sufficiently mature at the time of analysis. A formal assessment of interobserver reproducibility was not formally quantified (e.g., via intraclass correlation coefficients), although measurements followed a standardized protocol with multi-reader quality control and adjudication. In addition, detailed tumor burden metrics and endocrine sensitivity/resistance were not uniformly documented in this retrospective dataset, and residual confounding cannot be excluded. Finally, while median-based categorization was appropriate in the absence of validated adiposity thresholds, it may limit the generalizability of our findings, and alternative approaches (e.g., continuous modeling, external cutoffs, or validation cohorts) may be informative in future studies.

In conclusion, our findings add to accumulating evidence that baseline muscle mass is clinically meaningful in HR+/HER2− metastatic breast cancer treated with CDK4/6 inhibitors: non-sarcopenia and de novo metastatic presentation were independently associated with longer PFS, while adiposity indices and BMI were not prognostic in this cohort. These findings support routine assessment of skeletal muscle mass using baseline CT imaging to identify high-risk patients who may benefit from tailored supportive interventions. Prospective studies are warranted to determine whether interventions targeting muscle preservation can translate into improved clinical outcomes.

## Figures and Tables

**Table 1:** Baseline characteristics of the patients.

Parameters	*n* (%)	*p*
Age, years—median (IQR)	57.6 (48.4–67.6)	<0.05
<65	107 (68.6)	
≥65	49 (31.4)	
Menopausal status		<0.05
Postmenopausal	117 (75)	
Pre/perimenopausal	39 (25)	
Treatment line		>0.05
First line	80 (51.3)	
Second line	76 (48.7)	
Treatment regimen		>0.05
Ribociclib + Letrozole	67 (43)	
Ribociclib + Fulvestrant	50 (32)	
Palbociclib + Letrozole	17 (10.9)	
Palbociclib + Fulvestrant	22 (14.1)	
De novo metastasis		<0.05
No	106 (67.9)	
Yes	50 (32.1)	
ECOG-PS		>0.05
0	78 (50)	
1	78 (50)	
Sarcopenia		--
Yes	75 (48.1)	
No	81 (51.9)	
Metastasis site		>0.05
Bone-only	67 (42.9)	
Visceral	89 (57.1)	
BMI (kg/m^2^)—Mean (SD)	27.7 ± 5.0	>0.05
Normal weight (18.5–24.9)	49 (31.4)	
Overweight (25–29.9)	60 (38.5)	
Obese (≥30)	47 (30.1)	
Diabetes Mellitus		>0.05
Yes	53 (34)	
No	103 (66)	

**Abbreviations:** BMI, body mass index; ECOG-PS, Eastern Cooperative Oncology Group performance status; IQR, interquartile range; SD, standart deviation.

**Table 2:** Baseline body composition parameters.

Indices (cm^2^/m^2^)	Median	Mean ± Std. Deviation
SMA index	41.87	42.86 ± 8.52
SAT index	84.79	88.59 ± 37.39
VAT index	44.48	48.72 ± 29.85
IMAT index	4.72	5.71 ± 3.04
TAT index	134.99	142.92 ± 60.68

**Abbreviations:** IMAT, intermuscular adipose tissue; SAT, subcutaneous adipose tissue; SMA, skeletal muscle area; TAT, total adipose tissue; VAT, visceral adipose tissue.

**Table 3:** Univariable and multivariable Cox Regression analyses for progression-free survival.

	Univariable		Multivariable	
Variables	HR (95% CI)	*p*	HR (95% CI)	*p*
**Sarcopenia (ref: sarcopenic)**				
Non-sarcopenic	0.61 (0.41–0.91)	0.017	0.56 (0.37–0.85)	**0.006**
**De novo metastasis (ref: No)**				
Yes	0.55 (0.35–0.88)	0.013	0.58 (0.36–0.92)	**0.022**
**CDK4/6 inhibitor treatment (ref: Palbociclib)**				
Ribociclib	0.71 (0.46–1.01)	0.11	0.67 (0.43–1.03)	0.068
**Menopausal status** **(ref: postmenopausal)**				
Pre/perimenopausal	0.68 (0.41–1.11)	0.12	0.79 (0.45–1.39)	0.42
**ECOG-PS (ref: 1)**				
ECOG-PS: 0	0.72 (0.48–1.07)	0.11	0.68 (0.45–1.02)	0.063
**Metastasis site (ref: visceral)**				
Bone-only	0.75 (0.50–1.13)	0.17	0.79 (0.52–1.21)	0.28

**Abbreviations:** CDK4/6, cyclin-dependent kinase 4/6; ECOG-PS, Eastern Cooperative Oncology Group performance status; HR, hazard ratio; CI, confidence interval; ref, reference category.

## Data Availability

All original data generated or analyzed in this study are presented in this article. Additional information is available from the corresponding author upon request.
